# Supplementary material for: The Systems Biology Research Tool: evolvable open-source software
Source: BMC Syst Biol. 2008 Jun 29;2:55. doi: 10.1186/1752-0509-2-55 (PMC2446383; doi:10.1186/1752-0509-2-55)
Supplement: Additional file 1 — SBRT Archive. An archive of the current version of the Systems Biology Research Tool. [file 1752-0509-2-55-S1.zip › sbrt-1.4.0/doc/developers_guide/api/sbrt/shell/mng/ProcessManager.html]

ProcessManager


|  |  |  |
| --- | --- | --- |
| **Overview** \| **Package** \| **Class** \| **Use** \| **Tree** \| **Deprecated** \| **Index** \| **Help** | |  |
| **PREV CLASS**   **NEXT CLASS** | **FRAMES**    **NO FRAMES**     **All Classes** |  |
| SUMMARY: NESTED \| FIELD \| CONSTR \| METHOD | DETAIL: FIELD \| CONSTR \| METHOD |  |


---


## sbrt.shell.mng Interface ProcessManager<K,V>

**Type Parameters:**: `K` - the key type.: `V` - the value type.

**All Known Subinterfaces:**: KernelProcessManager<K,V>, ShellProcessManager<K,V>

**All Known Implementing Classes:**: AbstractProcessManager, BiggSbmlReaderManager, BiggSbmlTranslaterManager, CatalystDeletionManager, CdHarFluxVectorManager, CdHarManager, CorrelationManager, CvCatalystDeletionManager, CvFbaOptManager, CvMsFluxIntervalManager, CvObjectiveFunctionManager, CvRevRxnFluxIntervalManager, CvRxnDeletionManager, CvSimpleFluxIntervalManager, CycleIdManager, ExtremeCurrentManager, FbaManager, FbaOptManager, FbaSolverManager, FluxActivityManager, FluxCapManager, FluxPlasticityManager, InitialPointManager, IntervalComparisonManager, IntervalVariationManager, KendallTauManager, LinearCombConverterManager, LinearSolverManager, MannWhitneyUManager, MatrixConverterManager, MetatoolFileWriterManager, MmaNotebookManager, MsFluxIntervalManager, MsFluxomeReducerManager, MultipleVectorsConverterManager, NetworkInfoManager, ObjectiveFunctionManager, PalssonSbmlReaderManager, PalssonSbmlTranslaterManager, PathIdManager, RandomConstraintsManager, RandomEnvManager, RandomFluxVectorManager, RandomObjectiveManager, ReverseRxnFluxIntervalManager, RxnDeletionManager, RxnRemovalManager, SbmlWriterManager, SimpleFluxIntervalManager, SimpleRxnFileTransManager, SingleElementUnionManager, SingleVectorConverterManager, SlpCycleIdManager, StoichVariationManager, StrictSingleElementUnionManager, SvCdHarFluxVectorManager, SvInitialPointManager, UniqueCycleIdManager, VariableParticipationManager, VectorComparisonManager, WwFluxomeReducerManager

---

``` public interface ProcessManager<K,V> ```

This interface is used to represent process managers.
A process manager is used to manage a process executed by the Systems Biology
Research Tool.

A process manager is created from a `ManagerInput`
object. Process managers require a minimum set of key-value
pairs to perform their action.
In addition to this minimum set, a set of optional
key-value pairs may also be allowed. If the provided `ManagerInput`
contains any additional keys beyond the set of required and optional keys,
an `ApplicationException` will be thrown.

**Author:**
:   This interface was written and documented by
    Jeremiah Wright while in the Wagner lab.

---

| **Method Summary** | |
| --- | --- |
| `void` | `check(ManagerInput<K,V> input)`             Throws an `ApplicationException` if this `ProcessManager` cannot understand the provided input. |
| `void` | `execute()`             Executes the process this process manager controls. |
| `void` | `execute(java.io.PrintWriter out)`             Executes the process this process manager controls, and writes the status or results of this process to the provided print writer. |
| `java.util.Set<K>` | `getOptionalKeys()`             Returns the set of keys the provided `ManagerInput` object does not necessarily have to contain. |
| `java.util.Set<K>` | `getRequiredKeys()`             Returns the set of keys the provided `ManagerInput` object must contain. |
| `void` | `setInput(ManagerInput<K,V> input)`             Uses the provided input to prepare this process manager for execution. |
| `void` | `setInput(ManagerInput<K,V> input, java.io.PrintWriter out)`             Uses the provided input to prepare this process manager for execution. |

**Method Detail**

### getRequiredKeys

```
java.util.Set<K> getRequiredKeys()
```

:   Returns the set of keys the provided
    `ManagerInput` object must contain.

    :   **Returns:**: the set of keys the provided `ManagerInput` object must contain.

---


### getOptionalKeys

```
java.util.Set<K> getOptionalKeys()
```

:   Returns the set of keys the provided
    `ManagerInput` object does not necessarily
    have to contain.

    :   **Returns:**: the set of keys the provided `ManagerInput` object does not necessarily have to contain.

---


### setInput

```
void setInput(ManagerInput<K,V> input)
```

:   Uses the provided input to prepare this process
    manager for execution.

    :   **Parameters:**: `input` - all of the information required to prepare this process manager for execution.

---


### setInput

```
void setInput(ManagerInput<K,V> input,
              java.io.PrintWriter out)
```

:   Uses the provided input to prepare this process
    manager for execution. As the input is processed,
    informative messages are printed to the provided
    print writer.

    :   **Parameters:**: `input` - all of the information required to prepare this process manager for execution.: `out` - the print writer to which all messages will be printed.

---


### execute

```
void execute()
```

:   Executes the process this process manager controls.

    :   **Throws:**: `java.lang.IllegalStateException` - if no input was provided to this process manager via `setInput(ManagerInput)` or `setInput(ManagerInput, PrintWriter)`

---


### execute

```
void execute(java.io.PrintWriter out)
```

:   Executes the process this process manager controls,
    and writes the status or results of this process to the
    provided print writer.

    :   **Parameters:**: `out` - the print writer where some results or status messages will be printed. **Throws:**: `java.lang.IllegalStateException` - if no input was provided to this process manager via `setInput(ManagerInput)` or `setInput(ManagerInput, PrintWriter)`

---


### check

```
void check(ManagerInput<K,V> input)
```

:   Throws an `ApplicationException` if this `ProcessManager`
    cannot understand the provided input.

    :   **Parameters:**: `input` - the manager input to be checked.


---


|  |  |  |
| --- | --- | --- |
| **Overview** \| **Package** \| **Class** \| **Use** \| **Tree** \| **Deprecated** \| **Index** \| **Help** | |  |
| **PREV CLASS**   **NEXT CLASS** | **FRAMES**    **NO FRAMES**     **All Classes** |  |
| SUMMARY: NESTED \| FIELD \| CONSTR \| METHOD | DETAIL: FIELD \| CONSTR \| METHOD |  |


---
